# Application of a navigation system for contouring anatomical plasty of the distal end of the humerus

**DOI:** 10.3109/10929088.2012.692815

**Published:** 2012-06-08

**Authors:** Masayoshi Ikeda, Yuka Kobayashi, Ikuo Saito, Takayuki Ishii, Ayuko Shimizu, Yoshinori Oka

**Affiliations:** 1 Department of Orthopaedic Surgery, Tokai University Oiso Hospital, Kanagawa, Japan; 2 Department of Orthopaedics, Tokai University School of Medicine, Kanagawa, Japan; 3 Department of Orthopaedic Surgery, Tokai University Hachioji Hospital, Tokyo, Japan

**Keywords:** Navigation-assisted surgery, elbow joint, osteoarthritis, arthroplasty, anatomical plasty, osteophyte, coronoid fossa, olecranon fossa

## Abstract

The effectiveness of navigation systems in performing accurate orthopaedic surgery has been reported previously, but there have been no reports on the application of navigation in surgeries involving bone resection around the elbow joint. In this study, anatomical plasty or bone resection was performed to restore anatomical morphology in 10 cases of osteoarthritis of the elbow and deformity of the distal end of the humerus. Bone resection was performed on the distal end of the humerus using navigation and on the proximal end of the ulna via freehand surgery. Postoperatively, the elbow function was evaluated and pre- and postoperative CT images were used to measure the bone resection. There were no complications arising from the use of navigation, and elbow function was improved in all cases. By evaluating the CT images, it was found that navigated resection of the fossae of the distal humerus was more effective than freehand resection of the processes of the proximal ulna, thus confirming the usefulness of navigation. In future, to fully confirm this finding, it will be necessary to conduct prospective controlled studies of cases in which navigation is used to perform arthroplasty, including those that involve the proximal end of the ulna.

## Introduction

The elbow joint consists of the proximal ends of the radius and ulna and the distal end of the humerus. Various operative methods are used to treat traumas of the elbow joint such as fractures and ligament injuries, and diseases of the joint such as osteochon-dritis dissecans, osteoarthritis, and rheumatoid arthritis. These methods include open reduction and internal fixation, ligament reconstruction, arthroscopy, osteochondral grafts, debridement arthroplasty, and total elbow arthroplasty. However, minimally invasive approaches to surgery of the elbow joint are limited because of the nerves, blood vessels and muscles around the elbow. Occasionally, anterior and posterior approaches, medial and lateral approaches, and other approaches are used simultaneously to expose the elbow joint, depending on the surgical technique, the characteristics of the disease or trauma, and the morphological characteristics of the joint itself [1].

To visualize precisely the bone morphology of the elbow joint during surgery, it is necessary to evaluate preoperative computed tomography (CT) and three-dimensional (3D) CT images. Furthermore, there are some cases which require plain radiographs or the use of an image intensifier during surgery. However, the elbow joint is a morphologically unique structure. In particular, because the coronoid fossa and olecranon fossa are superimposed in a radiographic or image-intensified anteroposterior view of the distal humerus and the medial and lateral condyles are superimposed in a lateral view, it is often difficult to evaluate these forms from two-dimensional images [2, 3].

Therefore, a navigation system was used to perform elbow joint surgery to confirm the morphology of the distal end of the humerus. Because this was not a controlled study, the objective was not to demonstrate the superiority of the navigation-assisted surgery, but to describe a method for positioning the reference array and a registration technique for use with a navigation system at the distal end of the humerus. In addition, we assessed the usefulness of the navigation system as an effective tool for evaluating bony contours for the bone resection.

## Materials and methods

From April 2007 to December 2010, 9 patients at our institution with elbow osteoarthritis underwent open debridement arthroplasty to resect osteo-phytes, and one patient underwent contouring arthroplasty using a navigation system for deformity of the distal humerus. In each case the goal was to improve the bony morphology and elbow motion. A navigation system was used only during bone resection at the distal end of the humerus. All patients were subsequently followed for over 2 years after surgery. The study was approved by our institutional review board (study #10R-219).

All the patients were males, with an average age of 50 years (range: 15-74 years). In 5 patients the elbow osteoarthritis was attributed to long-standing participation in hard manual labor, and in 4 patients the osteoarthritis was caused by sports activities in which the patients had participated when young or in which they were currently active. One patient had an obliterated coronoid fossa of the distal humerus of unknown causality. All patients were informed about the planned use of the navigation-assisted surgery during the procedure and of the potential surgical complications, and all patients consented to the use of the navigation system.

In each case, a CT scan was performed before surgery. An Aquilion 64 CT scanner (Toshiba, Tokyo, Japan) was used, and the scan was performed with 0° of gantry angle, 0.6-mm slice thickness, and a soft tissue contrast sharp setting. These CT data were later used with a CT-based navigation system. The bone morphology, including osteophytes in the distal humerus or proximal forearm, was evaluated and bone resection was performed to restore the normal anatomical morphology; we refer to these procedures as “anatomical plasty". The amount of bone to be resected, including osteophytes, was determined from preoperative CT images with the goal of achieving normal anatomical bone morphology. Drill burrs were used in conjunction with a navigation system to resect osteophytes of the coronoid, radial, and olecranon fossae of the distal end of the humerus. In contrast, chisels were used for freehand resection of osteophytes of the olecranon and coronoid processes under direct vision without the use of a navigation system.

The average postoperative follow-up period was 29 months (range: 24-36 months).

### Navigation-assisted surgery in anatomical plasty

A CT-based navigation system (VectorVision, BrainLAB, Heimstetten, Germany) developed for spinal surgery was used to perform the navigation-assisted bone resection at the distal end of the humerus. The spinal navigation system was applied with the consideration that the distal dorsal side of the humerus was morphologically similar to the posterior aspect of the vertebral lamina. To register the distal humerus, a total of 4 points, including the medial and lateral epicondyles and two points on the margin of the olecranon fossa, were used as global orientation landmarks. These 4 points were uploaded into the navigation workstation together with the preoperative CT images and matched with the actual anatomical position of the distal humerus in the registration process during surgery.

Patients were placed in the supine position under general anesthesia, the shoulder was held in the 90° abducted position, and a medial approach to the elbow joint was performed. Sterilized pneumatic tourniquets were used during surgery. The anterior and posterior sides of the elbow joint were exposed, with the inter-muscular septum between the bra-chial and triceps muscles serving as the boundary.

**Figure 1:**
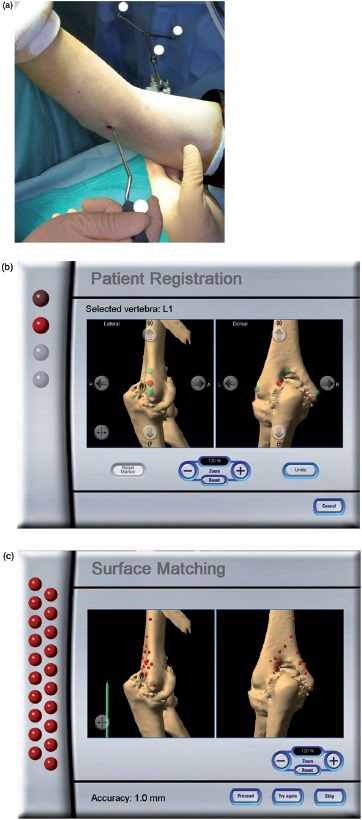
Registration and surface matching, (a) A reference array was placed on the medial side of the humerus. (b) A global registration was performed using 4 points, including the medial and lateral epicondyles and the two points on the edge of the olecranon fossa. Since the navigation software was designed for use in spinal surgery, the distal humerus was designated as vertebra LI, as shown in the screen shot, (c) Surface matching was then performed with 20 points on the posterior aspect of the distal humerus. The term “Accuracy: 1.0 mm” visible at the bottom of this screen was automatically displayed by the system.

**Figure 2:**
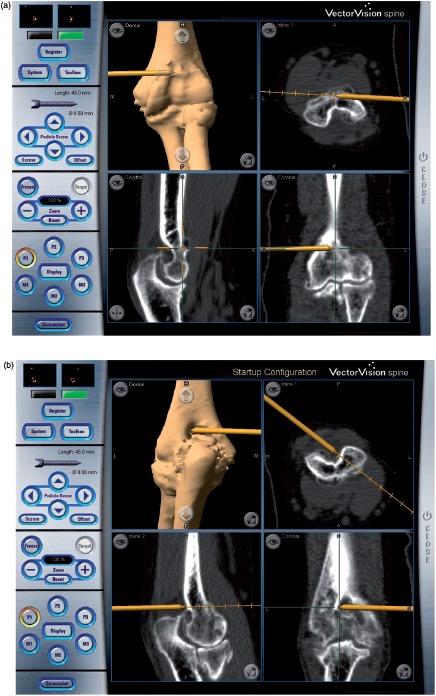
Navigation-assisted surgery. A drill burr with a mounted reference array is used for bone resection of the distal humerus. (a) Anterior side of the joint. The drill burr is shown as a gold rod that is drilling out the osteophyte on the coronoid fossa, (b) Posterior side of the joint. The drill is burring out the olecranon fossa.

The reference array of the navigation system was set perpendicular to the medial cortex of the humerus, anterior to the ulnar nerve, and approximately 10 cm proximal to the medial epicondyle. When the reference array was set in this position, the elbow joint could be extended and flexed freely, and an anterior or posterior approach was possible with the shoulder in the 90° abducted position and the elbow in a flexed or extended position. Initially, the medial and lateral epicondyles and the two points on the margin of the olecranon fossa were registered with a straight pointer (Figure la and b). Next, surface matching was performed with 20 points on the posterior aspect of the distal humerus (Figure 1c). By means of this registration process, navigation of the entire surface of the distal humerus was possible. The accuracy of the navigation system averaged 1.1mm (0.6-2.1 mm) according to the navigation workstation. Next, the other reference array was mounted on the straight-type drill with a 4-mm steel burr (Command Micro Components, Stryker, Kalamazoo, MI) and registration was performed. This drill burr was used in the procedure to form the original anatomical contour of the humerus on the navigation workstation (Figure 2). On the posterior side of the elbow joint, the bone on the posterior surface of the distal humerus, including osteophytes in the olecranon fossa, was resected. On the anterior side of the joint, the bone around the radial fossa and the coronoid fossa on the anterior surface of the distal humerus was resected between the posterior edge of the distal brachial muscle and the flexor-pronator muscle origin attached to the humerus. In contrast, a chisel was used under direct vision for freehand resection and contouring of the bone of both the olecranon and coronoid processes to form the anatomical morphology.

In all cases, CT imaging with 0.6-mm slices was performed after surgery. By comparing the pre- and postoperative CT images, a determination was made as to whether the anatomical-morphological contour could be formed and reproduced in the distal part of the humerus and proximal part of the ulna, according to the preoperative goal.

**Figure 3:**
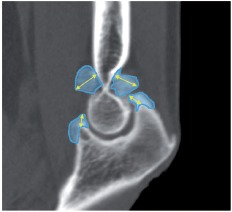
Measurement of bone morphology. A CT image of a sagittal plane through the bottoms of the coronoid and olecranon fossae of the distal humerus. The maximal values of the heights of the bone protruding from the contour surface, which is assumed to be the normal anatomical morphology, are shown. The heights of the bones (yellow lines with arrows) and their areas (in blue) are measured.

CT images of the sagittal plane through the bottoms of the coronoid and olecranon fossae were used. An image software package (Adobe Photoshop CS4, Adobe Systems, San Jose, CA) was used to measure the maximum heights and areas of the bones projecting from the normal anatomical contour in the coronoid and olecranon fossae and the olecranon and coronoid processes (Figure 3). The pre- and postoperative values of the height and area of the bones in each of the parts were compared. At the same time, the corresponding heights and areas of the bone remaining post-operatively were compared with each other at the sites operated with and without use of the navigation system, and assessed to determine whether the preoperatively assumed normal morphological contour was achieved in bone resection. The following formula was used to calculate the ratio of the resected bone area to the preoperatively projected bone area:


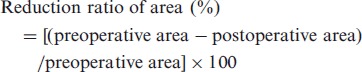


The range of motion of the elbow joint was measured to determine the elbow function in the preoperative period and during follow-up. The Mayo Elbow Performance Score (MEPS) was used to evaluate the elbow joint function [4]. Furthermore, the Disabilities of the Arm, Shoulder and Hand questionnaire form (DASH score) was used to evaluate the subjective status of the arm function.

### Statistical analysis

In each case, the Wilcoxon signed-rank test was used to compare the ranges of motion of the elbow joint, MEPS, and DASH scores in the preoperative and postoperative follow-up periods. Moreover, the Kruskal-Wallis test and Tukey's honestly significant difference test were used to compare the measurement values for the bone in the olecranon and coronoid processes and the coronoid and olecranon fossae. Differences with p < 0.05 were considered statistically significant.

## Results

During surgery, the distal end of the humerus could be well navigated on the workstation screen, which, in addition to direct vision, facilitated bone resection by ensuring a correct burring-out depth in each case. On clinical examination, satisfactory results were observed in all 10 cases. The ranges of motion of the elbow joints were improved in all cases; in particular, elbow flexion was improved significantly from 107.8° to 127.7°. Significant improvements were observed in the MEPS (from 76.5 to 98.5 points) and in the DASH score (from 25.6 to 8.9 points) (Table I).

**Table I:** Patient data. Values are expressed as mean ± standard deviation with the range in parentheses.

		Pre-op	Post-po	Wilcoxon signed-rank test
ROM of elbow (°)	Extension	-16± 11.9 (-36-12)	-12±4.3 (-19--5)	p = 0.074
	Flexion	107.8 ±18.9 (70-126)	127.7±4.4 (118- -134)	p = 0.005
MEPS* (point)		76.5 ±9.1 (65-85)	98.5 ±2.4 (95- 100)	p = 0.005
DASH score+ (point)		25.6 ±9.1 (12.5-38.6)	8.9±8.3 (1.6-- 24.0)	p = 0.011

* MEPS = Mayo Elbow Performance Score.

+ DASH = Disabilities of the Arm, Shoulder and Hand.

There was a significant size reduction in all values postoperatively when compared with the preoperative values measured on the CT images at each site (p<0.05). In the coronoid and olecranon fossae, where navigation was used, the height of the remaining bone was significantly reduced compared with that of the olecranon and coronoid processes of the proximal ulna, where navigation was not used to perform the freehand resection. The percentage of the resection with regard to the preoperatively projected area was more than 90% in the coronoid and olecranon fossae, and significantly greater resection could be achieved compared with that of the olecranon and coronoid processes (Table II). During surgery, and throughout the postoperative period, there were no complications such as neurovascular injuries or bone fractures in any of the patients as a result of using navigation.

**Table II:** Morphometric measurement of osteophytes. Values are expressed as mean ± standard deviation (range in parentheses). The height and area of the osteophyte and the reduction ratio of the osteophyte area were compared pre- and postoperatively.

		Pre-op†	Post-op†	Reduction ratio of Area* (%)	Tukey's HSD test
Height of osteophyte (mm)	Coronoid fossa	6.4 ±1.4 (4.7−9.5)	2.1 ± 1.1 (0.1−3.7)^a^		^a^p = 0.065
	Olecranon fossa	7.2 ±2.3 (3.8−10.5)	1.4±0.5 (0.8−2.0)^b^'^c^		^b^p = 0.014
	Coronoid process	5.5±2.1 (3.2−9.7)	3.l±0.7 (1.4−4.1)^b^		^c^p = 0.003
	Olecranon process	5.5±1.6 (2.8−8.9)		3.4±1.5 (1.0−5.7)^a^'^c^
Area of osteophyte (mm^2^)	Coronoid fossa	60.6 ± 28.4 (21.4−113.4)	12.3 ± 6.8 (3.8−25.7)	95.9±2.5 (91.2−99.6)^d,e^	^d^p = 0.011
	Olecranon fossa	58.6±33.2 (13.6−105.1)	7.5±3.9 (1.2−12.9)	95.8±3.7 (87.0−99.2)^f,g^	^e^p = 0.001
Coronoid process	31.7±12.0 (19.2 −56.0)	11.7±4.2 (6.4−18.9)	88.9 ±5.2 (79.2−94.0)^d,f^	^f^p = 0.015
Olecranon process	29.6 ±14.5 (16.6−54.5)	13.1±6.7 (3.7−25.6)	87.2±6.4 (78.3−94.0)^e,g^	^g^p = 0.002

* Reduction ratio of area = [(preoperative area — postoperative area)/preoperative area] x 100.

† All values were significantly reduced postoperatively compared to preoperative values (p< 0.05).

Degrees of reduction (height, area) were compared to one another using Tukey's HSD test followed by a Kruskal-Wallis test (right end column).

## Discussion

In previous reports on anatomical plasty of bones to restore the normal bone morphology of the elbow joint, such as those in which debridement arthro-plasty was used [5–9], the authors believed that great reliance had to be placed on the surgeon's experience and spatial sense to precisely grasp the geometric relationships of the joint, even when image intensification and radiographs were used during surgery [2, 3]. This was because few parts of the elbow joint are formed by flat planes, implying that the entire image of the joint cannot be observed directly through a single small-incision approach, and that it is difficult to capture the full aspect of the bony structure of the elbow joint from each approach. Therefore, in this study, a navigation system was employed to provide real-time feedback on the bone morphology and enable real-time tracking of the surgical instruments. The goal of navigation-assisted surgery in the field of orthopedic surgery is to obtain maximal accuracy with minimal invasiveness [10–12]. Navigation systems have been used in many orthopedic procedures, such as ligament reconstruction of the knee [13, 14], total joint arthroplasties of the hip and knee [15], pedicle screw insertion in spinal surgery [16, 17], and trauma surgery [18], and have been reported to be useful. However, there have been almost no reports of their clinical application in elbow joint surgery until now [19].

The purpose of this study was to assess the application of a navigation system in bone resections around the elbow, but because evaluation was limited to bone resection of the distal end of the humerus, there were great limitations as regards evaluating its usefulness. Reasons for using navigation only for the distal end of the humerus were that the reference array could be easily installed on the humerus, and that it was easy to use the posterior surface of the distal end of the humerus for registration. Another reason was that it has been more difficult in clinical situations to evaluate the morphology in resections of concave surfaces of the fossae of the distal humerus, as compared to evaluating bone resection at the tip of the process of the proximal ulna.

In this study, for quantitative assessment of osteophyte size and the amount of bone resected, the anatomical morphologic contours on CT images of a sagittal plane passing through the bottoms of the coronoid and olecranon fossae of the distal humerus were used to measure the heights and areas of the bones. These sites were also correlated with the tops of the olecranon and coronoid processes of the proximal ulna.

Other limitations that should be mentioned are those of navigation errors arising from the calibration of drill burrs with large diameters, errors produced by the surgeon's freehand use of surgical instruments with reference arrays attached, and the surgeon's visualization errors encountered when using the navigation system [20, 21].

Nevertheless, we conclude that the use of a navigation system in bone resection at the distal end of the humerus significantly improved visibility and monitoring compared with that in freehand resection of the proximal end of the ulna, and that navigation-assisted surgery is reliable and useful, even considering the differences in accessibility or ease of identifying reference landmarks. We believe that in practical clinical situations where anatomical plasty is required, it is desirable to resect a greater quantity of bone rather than reproduce the original morphological contour.

On the other hand, the greatest drawback of navigation-assisted surgery is the necessity of fixing the reference arrays on the bone and the surgical instruments [22]. In this patient series, the reference array was set up on the medial side of the humerus just anterior to the ulnar nerve. The reasons for this were that the patient was supine, and the anterior and posterior sides of the elbow joint could be exposed via both medial and lateral approaches without obstruction of elbow motion by the reference array or injury to the nerves and blood vessels, while the posterior surface of the distal humerus was used for registration. Navigation-assisted surgery is not a minimally invasive procedure, but there were no intraoperative complications caused by the use of navigation in this series. However, it is troublesome to position the patient's arm and the surgical instrument so that the 3D optical localizer can fully recognize the reference array during surgery.

This series involved navigation-assisted surgery only on the distal end of the humerus. To ascertain the practicality of this approach, it will be necessary to apply navigation to the proximal part of the ulna and to perform prospective controlled studies of surgeries with and without the use of navigation systems. Future improvements should attempt to enable the use of navigation in combination with arthroscopic surgery of the elbow.

## References

[1] Morrey BF, Morrey BF (2009). Surgical exposures of the elbow. The Elbow and Its Disorders.

[2] Oka Y, Ohta K, Saitoh I (1998). Debridement arthroplasty for osteoarthritis of the elbow. Clin Orthop Relat Res.

[3] Oka Y (2000). Debridement arthroplasty for osteoarthritis of the elbow: 50 patients followed mean 5 years. Acta Orthop Scand.

[4] Morrey BF, Morrey BF (2009). Functional evaluation of the elbow. The Elbow and Its Disorders.

[5] Tsuge K, Mizuseki T (1994). Debridement arthroplasty for advanced primary osteoarthritis of the elbow. Results of a new technique used for 29 elbows. J Bone Joint Surg Br.

[6] Phillips NJ, Ali A, Stanley D (2003). Treatment of primary degenerative arthritis of the elbow by ulnohumeral arthro plasty. A long-term follow-up. J Bone Joint Surg Br.

[7] Sarris I, Riano FA, Goebel F, Goitz RJ, Sotereanos DG (2004). Ulnohumeral arthroplasty: Results in primary degenerative arthritis of the elbow. Clin Orthop Relat Res.

[8] Wada T, Isogai S, Ishii S, Yamashita T (2004). Debridement arthroplasty for primary osteoarthritis of the elbow. J Bone Joint Surg Am.

[9] Tashjian RZ, Wolf JM, Ritter M, Weiss AP, Green A (2006). Functional outcomes and general health status after ulno humeral arthroplasty for primary degenerative arthritis of the elbow. J Shoulder Elbow Surg.

[10] Hazan EJ (2003). Computer-assisted orthopaedic surgery. A new paradigm. Techniques in Orthopaedics.

[11] DiGioia AM, Blendea S, Jaramaz B (2004). Computer-assisted orthopaedic surgery: Minimally invasive hip and knee reconstruction. Orthop Clin North Am.

[12] Hafez MA, Seel MJ, Jaramaz B, DiGioia AM (2006). Navigation in minimally invasive total knee arthroplasty and total hip arthroplasty. Oper Tech Orthop.

[13] Mauch F, Apic G, Becker U, Bauer G (2007). Differences in the placement of the tibial tunnel during reconstruction of the anterior cruciate ligament with and without computer- assisted navigation. Am J Sports Med.

[14] Picard F, DiGioia AM, Moody J, Martinek V, Fu FH, Rytel M, Nikou C, Labarca RS, Jaramaz B (2001). Accuracy in tunnel placement for ACL reconstruction. Comparison of tradi tional arthroscopic and computer-assisted navigation tech niques. Comput Aided Surg.

[15] Amiot LP, Poulin F (2004). Computed tomography-based naviga tion for hip, knee, and spine surgery. Clin Orthop Relat Res.

[16] Merloz P, Tonetti J, Pittet L, Coulomb M, Lavallee S, Troccaz J, Cinquin P, Sautot P (1998). Computer-assisted spine surgery. Comput Aided Surg.

[17] Merloz P, Huberson C, Tonetti J, Eid A, Vouaillat H (2003). Computer-assisted pedicle screw insertion. Techniques in Orthopaedics.

[18] Kendoff D, Citak M, Hiifner T, Chaudhary S, Krettek C (2007). Current concepts and applications of computer navigation in orthopedic trauma surgery. Central European Journal of Medicine.

[19] McDonald CP, Johnson JA, Peters TM, King GJ (2010). Image- based navigation improves the positioning of the humeral component in total elbow arthroplasty. J Shoulder Elbow Surg.

[20] Arand M, Schempf M, Fleiter T, Kinzl L, Gebhard F (2006). Qualitative and quantitative accuracy of CAOS in a stan dardized in vitro spine model. Clin Orthop Relat Res.

[21] Phillips R (2007). The accuracy of surgical navigation for orthopaedic surgery. Current Orthopaedics.

[22] Zheng G, Kowal J, Gonzalez Ballester MA, Caversaccio M, Nolte LP (2007). Registration techniques for computer navigation. Current Orthopaedics.

